# High-Throughput Field Imaging and Basic Image Analysis in a Wheat Breeding Programme

**DOI:** 10.3389/fpls.2019.00449

**Published:** 2019-04-24

**Authors:** James Walter, James Edwards, Jinhai Cai, Glenn McDonald, Stanley J. Miklavcic, Haydn Kuchel

**Affiliations:** ^1^School of Agriculture, Food and Wine, The University of Adelaide, Glen Osmond, SA, Australia; ^2^Australian Grain Technologies Pty Ltd., Roseworthy, SA, Australia; ^3^Phenomics and Bioinformatics Research Centre, School of Information Technology and Mathematical Sciences, University of South Australia, Mawson Lakes, SA, Australia

**Keywords:** phenotyping, physiological yellows, senescence, septoria tritici blotch, canopy cover

## Abstract

Visual assessment of colour-based traits plays a key role within field-crop breeding programmes, though the process is subjective and time-consuming. Digital image analysis has previously been investigated as an objective alternative to visual assessment for a limited number of traits, showing suitability and slight improvement to throughput over visual assessment. However, easily adoptable, field-based high-throughput methods are still lacking. The aim of the current study was to produce a high-throughput digital imaging and analysis pipeline for the assessment of colour-based traits within a wheat breeding programme. This was achieved through the steps of (i) a proof-of-concept study demonstrating basic image analysis methods in a greenhouse, (ii) application of these methods to field trials using hand-held imaging, and (iii) developing a field-based high-throughput imaging infrastructure for data collection. The proof of concept study showed a strong correlation (*r* = 0.95) between visual and digital assessments of wheat physiological yellowing (PY) in a greenhouse environment, with both scores having similar heritability (*H^2^* = 0.85 and 0.76, respectively). Digital assessment of hand-held field images showed strong correlations to visual scores for PY (*r* = 0.61 and 0.78), senescence (*r* = 0.74 and 0.75) and Septoria tritici blotch (STB; *r* = 0.76), with greater heritability of digital scores, excluding STB. Development of the high-throughput imaging infrastructure allowed for images of field plots to be collected at a rate of 7,400 plots per hour. Images of an advanced breeding trial collected with this system were analysed for canopy cover at two time-points, with digital scores correlating strongly to visual scores (*r* = 0.88 and 0.86) and having similar or greater heritability. This study details how high-throughput digital phenotyping can be applied to colour-based traits within field trials of a wheat breeding programme. It discusses the logistics of implementing such systems with minimal disruption to the programme, provides a detailed methodology for the basic image analysis methods utilized, and has potential for application to other field-crop breeding or research programmes.

## Introduction

Visual assessment of traits within field trials is subjective and laborious. However, it is an essential process for plant breeders who wish to observe the phenotype of material within their programme and determine genotype-by-environment effects. In recent years numerous high-throughput digital phenotyping methods have been proposed (Busemeyer et al., 2013; White and Conley, 2013; Andrade-Sanchez et al., 2014; Deery et al., 2014; Bai et al., 2016; Underwood et al., 2017; Jimenez-Berni et al., 2018), all of which offer to alleviate the current visual phenotyping bottleneck which exists within modern plant breeding programmes (Cobb et al., 2013; Araus and Cairns, 2014). Despite this, truly high-throughput systems which are easily integrated within large-scale breeding programmes are yet to be developed and used.

Typically, these phenotyping platforms are equipped with an array of sensors, with popular choices including red, green and blue (RGB) cameras, multi-spectral cameras, normalised difference vegetation index (NDVI) sensors and LiDAR. RGB cameras, in particular, have a long history with field phenotyping and in a number of studies have been effective in estimating canopy cover of field crops (Lukina et al., 1999; Casadesús et al., 2007; Liu and Pattey, 2010; Mullan and Reynolds, 2010). The popularity of these methods, from both a research and farmer perspective, has culminated in the development of a simple mobile application, which enables users to conduct simple *in-situ* estimates of canopy cover from their mobile devices (Oklahoma State University, 2015). The use of RGB cameras as a phenotyping tool has focused on digital images to estimate canopy cover or as an alternative to NDVI (Casadesús et al., 2007; Morgounov et al., 2014). However, they have also been used to a lesser extent to assess senescence (Adamsen et al., 1999; Hafsi et al., 2000), crop nitrogen content (Li et al., 2010), early vigour (Kipp et al., 2014) and soil water evaporation (Mullan and Reynolds, 2010). Image analysis techniques used to asses this range of traits also have the potential to be applied to other colour-based traits, such as disease assessment, which may provide wheat breeders with an objective system of assessment for specific traits within their breeding programme.

In the current study, we collected data on four traits [physiological yellowing (PY), senescence, Septoria tritici blotch (STB), and canopy cover] from within a Southern Australian bread wheat breeding programme, using high-throughput image collection and basic, open-source, image analysis.

Physiological yellowing of bread wheat (*Triticum aestivum* L) and durum wheat (*T. durum*) can have a number of possible causes, however, there is little literature surrounding the trait, with only a single study and two industry fact sheets exploring the effect (Australian Grain Technologies, 2013, 2016; Schwenke et al., 2015). Further to the reported yield impacts, farmer perception often marks material expressing PY as undesirable, due to its “disease-like” symptoms.

Senescence is yellowing of green leaves and the eventual browning and drying of leaf material as a crop matures. Senescence occurs naturally with time and can be used as indicator of maturity or the impact of abiotic stress (Distelfeld et al., 2014).

Septoria tritici blotch is a foliar disease of wheat due to infection by the fungus *Zymoseptoria tritici*. Resistance for STB is actively sought within breeding programmes (Brown et al., 2015). Expression of STB is observed as yellow/brown lesions on leaves, containing small black fruiting bodies (pycnidia). Assessment of STB in breeding programmes typically occurs in inoculated disease nurseries to ensure there is adequate incidence of the disease.

Canopy cover is the proportion of soil covered by the crop canopy, and is primarily used for the assessment of early vigour. It is associated with the reduction of soil water evaporation (Rebetzke et al., 2004; Mullan and Reynolds, 2010) and weed competitiveness (Lemerle et al., 1996; Coleman et al., 2001). In a more crude form it is also used to identify plant establishment issues in field trials.

While each of the traits investigated in the current study is physiologically different, they are linked through the colour-based nature of their visual assessment. Visual assessment for each of these traits is typically achieved through either a percentage score, or through a 1–9 scale of severity. This type of assessment lends itself to the application of image analysis, where percentage area within images can be calculated.

The aim of the current study was to develop a high-throughput digital imaging system capable of assessing colour-based traits observed within a wheat breeding programme. This was achieved in three stages:

(i) A proof of concept study in a greenhouse to develop a method and examine how effectively freely available image analysis software and consumer digital cameras can estimate colour-based traits.
(ii) Applying these concepts of hand-held digital imaging and basic image analysis to field trials to demonstrate their application in breeding programmes.
(iii) Using the results of i and ii to develop a field-based high-throughput imaging infrastructure, with a basic image analysis pipeline.

The first two years of the current study involved the development and testing of data capture and processing systems, and the third year tested these systems within a wheat breeding field trial.

## Materials and Methods

The three stages of the current study were conducted during the seasons of 2015, 2016, and 2017 using a combination of hand-held and high-throughput RGB imaging in greenhouse and field trials (Table 1). Images were collected opportunistically within a large-scale wheat breeding programme, across seven experiments, for PY, senescence, STB and canopy cover. While multiple traits were observed in the field, it is proposed that the image analysis methods can be applied to any colour-based trait of interest.

**Table 1:** Summary of trials assessed in the current study.

Trial	Environment	Location	Position	Trait Measured	Observations (*n*)	Replicates
*Greenhouse*	*Controlled*	Roseworthy	34°31’58.40”S, 138°41’20.60”E	Physiological Yellows	72 (plants)	3
*A*	*Field*	Roseworthy	34°30’33.51”S, 138°40’26.03”E	Physiological Yellows	432	2
*B*	*Field*	Roseworthy	34°30’33.51”S, 138°40’26.03”E	Physiological Yellows	432	2
*C*	*Field*	Roseworthy	34°30’33.51”S, 138°40’26.03”E	Senescence	240	Partial (25%)
*D*	*Field*	Roseworthy	34°30’33.51”S, 138°40’26.03”E	Senescence	648	Partial (25%)
*E*	*Field*	Turretfield	34°32’13.81”S, 138°50’36.55”E	Septoria Tritici Blotch	202	2
*F*	*Field*	Winulta	34°15’12.41”S, 137°53’3.21”E	Canopy Cover	288	3

### Greenhouse Imaging

Imaging of a potted experiment investigating the expression of PY was conducted to establish the feasibility of assessing a colour-based trait with basic open-source image analysis methods. The experiment consisted of individually potted plants arranged in a randomised block design of three replicates, with treatments of genotype and presence/absence of chlorine (Cl^-^) as described by Schwenke et al. (2015). Plants were grown in a greenhouse on the University of Adelaide, Roseworthy Campus. Further details of this experiment are described by Australian Grain Technologies (2016).

The severity of symptoms was assessed shortly after anthesis [Zadoks Growth Scale 69 (Z69) (Zadoks et al., 1974)], as the percentage of leaf area affected by PY, i.e., a visual estimate of the percentage of leaf material that was yellow. To obtain image analysis scores, RGB images were captured for every plant using a commercial digital camera (Canon 100D) at a resolution of 3456 × 5184 pixels (18 MP), with auto exposure. Plants were placed in front of a white background, to allow for simplified image processing and analysis. Images were captured from the side of pots, allowing for large amount of leaf area to be visible, with minimal occlusion.

### Field Imaging

Following the testing of imaging in the potted greenhouse experiment, imaging methods were adapted and deployed within six wheat breeding field trials which examined a number of different traits (Table 1). Field plot trials consisted of small plots 1.32 m × 3.2 m (trials A–D, F) or 0.45 m × 1 m (trial E) in size, with each trial containing a single treatment of genotype, with varying levels of replication (Table 1), arranged in a completely randomised design. Field plots were managed by Australian Grain Technologies (AGT) within their wheat breeding programme, with plots in trial E grown within an inoculated STB nursery.

Visual and digital scores were recorded as percentage of yellow leaf area, with visual scores collected in the field following imaging. Exceptions to this were trial E, where visual STB severity was assessed using a 1:9 scale at the time of imaging and trial F, where visual scores were recorded as percentage canopy cover obtained by a visual estimate of canopy cover in individual images, and digital scores were calculated as the percentage of image area that was green.

Images of plots in trials A–E were captured at a nadir angle by using a hand-held camera (Canon 100D) over each plot, approximately 1.5 m above ground level. Images were captured at a resolution of 3456 × 5184 pixels (18 MP), with exposure settings adjusted *ad-hoc*. Nadir images were chosen because lateral images reveal only the first few plants in each row, with the rest of the plot being occluded. Images of plots in trial F were collected at a nadir angle using the High-throughput Imaging Boom (HIB; described in detail below). Plots were imaged early in the season (approximately Z25) and following anthesis (approximately Z69) to observe plot establishment and canopy cover. Images were captured automatically using the HIB at both time-points, with cameras set to 1/1000 and 1/2000 of a second shutter speed at the first and second time-points, respectively, *f* 8.0 aperture and auto ISO to allow for exposure compensation.

### Image Analysis

All images were processed in the FiJI distribution (Schindelin et al., 2012) of the open-source software ImageJ (Schneider et al., 2012), using the Threshold Colour plugin (Supplementary Data 1). Central regions of interest were applied to greenhouse images and field images, where plant material did not fill the frame.

**FIGURE 1:**
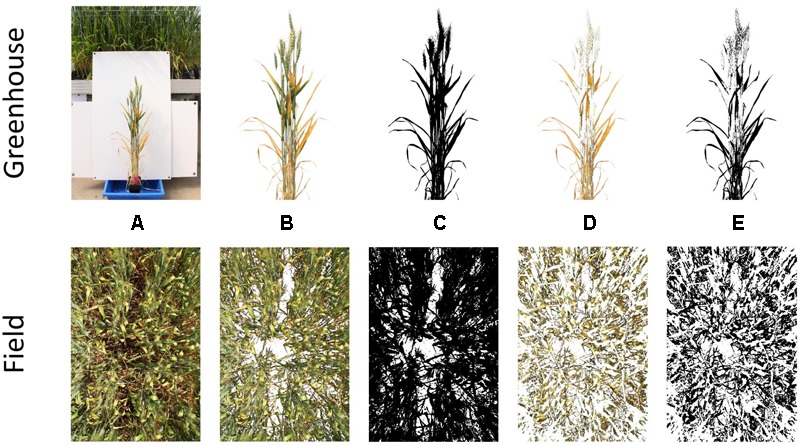
Stages of the thresholding process for single plants grown in the greenhouse (top) and whole plots grown in the field (bottom). Depicted are the original image **(A)**, segmented plant material **(B)**, binary plant material threshold **(C)**, segmented yellow material **(D)**, and binary yellow material threshold **(E)**.

A two-stage thresholding process was then used to separate firstly, all plant material from background material (i.e., white corflute in greenhouse and soil in field), and secondly yellow plant material from green. Examples of this process are shown for PY in Figure 1. Yellow thresholding was not required for the estimation of canopy cover. Thresholding was conducted using Hue, Saturation and Brightness (HSB) values, with these being visually determined for each experiment to obtain the most suitable thresholds. Once threshold images had been created, the number of plant material pixels and yellow pixels were counted, allowing the percent yellow leaf area score (or percent image area green) to be calculated. Detailed methods for thresholding and batch processing of images are available in Supplementary Data 1. Examples of processed images for senescence, STB and canopy cover are available in Supplementary Data 2.

Images obtained from field trials were resized to 25% of their longest edge (∼1 MP), to increase processing speed and avoid RAM limitations, when batch processing large numbers of images.

### High-Throughput Imaging Boom Development

**FIGURE 2:**
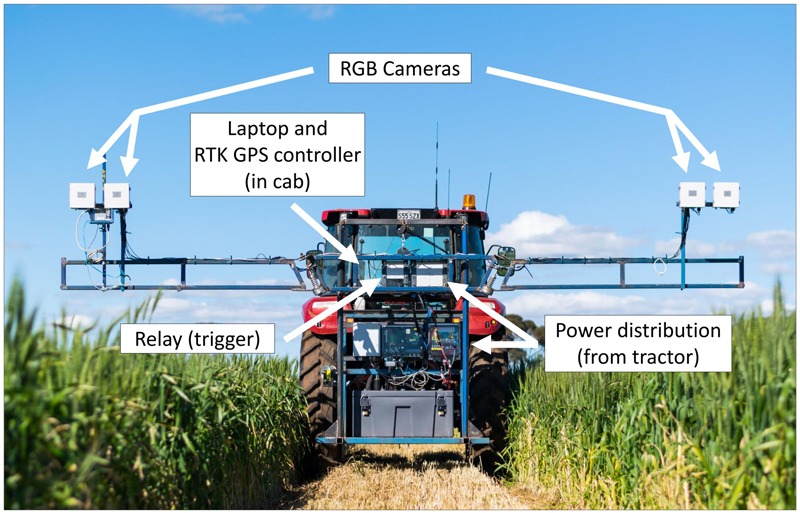
The High-throughput Imaging Boom (HIB) developed and deployed as part of the current study, parked in a maintenance pathway of a wheat field trial. Annotations outline major components of the system.

The High-throughput Imaging Boom shown in Figure 2 was designed for the express purpose of integration into a large-scale wheat breeding programme. It features four commercially available digital cameras (Canon 70D) mounted inside weather sealed boxes on the boom arms. This setup allows two images per plot to be captured simultaneously, and the potential for future work to investigate applications of stereo imaging.

Image capture is triggered by a single relay, which is controlled by a laptop computer in the tractor cab. The laptop uses proprietary software to monitor GPS output from a Trimble FM1000 RTK GPS unit and trigger the relay from a set of predefined GPS coordinates, camera trigger delay and the distance between GPS receiver and the cameras. GPS coordinates are computed based on three corner coordinates of the trial site and the number of plot rows and columns present at the site. The HIB is driven to each of these three corners and the cameras positioned over the end plot. Once in position the GPS coordinates are saved within the software. After collecting the three GPS coordinates, individual triggering coordinates for each plot are interpolated from the three corner positions. A text file containing all trigger coordinates is saved and can be loaded into the software for every imaging event, meaning this setup process need only be completed once per field site.

The boom on which the cameras are mounted features arms of adjustable height, which fold in for transport, mimicking a standard spray boom used for plot maintenance within the wheat breeding programme. To further strengthen the concept of integrating the HIB within a field-crop breeding programme, the tractor to which it is attached can use the GPS autosteer function of the RTK GPS unit, adhering to the predefined maintenance pathways within the trial. These pathways are typically used for standard management practises such as fertiliser, herbicide and fungicide application. This reduces operator error while driving the tractor, and allows repeated image capture throughout the season with a spatial accuracy of 2 cm. To operate the HIB the tractor is driven down maintenance pathways within each field trial, with boom arms placing the cameras centrally over one plot each side of the tractor.

As the tractor drives, image capture occurs automatically, with images stored on SD cards within individual cameras. The software running on the laptop computer monitors GPS message output from the RTK GPS unit, with this information being used to determine triggering of the relay in conjunction with the pregenerated trigger coordinate text file. This process accounts for tractor speed (calculated from GPS coordinates), signal travel time between laptop and camera shutter trigger (predefined within the software) and the distance between the GPS receiver and cameras. The tractor continues to travel along maintenance pathways in a serpentine manner, until all plots have been imaged.

The HIB was driven at 5 km/h during image capture. Cameras had manually set exposures, with a shutter speed of 1/1000 or 1/2000 of a second (for images at Z25 and Z69, respectively), an aperture of *f* 8.0 and auto ISO to allow for exposure compensation. Images were captured in JPEG format for ease of post processing, and because of limitations in image write speed and buffer capacity of the cameras for RAW images.

### Statistical Analysis

All statistical analysis was conducted in the R software package (R-Core Team, 2017). Mixed linear models were used to analyse all data sets through univariate and bivariate analyses of visual and digital measurements using ASReml (Butler et al., 2009). Pearson’s correlations between raw data were calculated within univariate analyses, while genetic and residual correlations were calculated from bivariate analyses. Broad-sense trait heritability (Eq. 1), which can be described as the proportion of observed trait variation attributable to genetics (Visscher et al., 2008), was also calculated within univariate analyses.

(1) $$H^{2}=\;\frac{\sigma_{G}^{2}}{\sigma_{G}^{2}+\;\;\sigma_{E}^{2}\;}$$

where H^2^ is broad-sense heritability, $\sigma_{G}^{2}$ is the variance attributable to genetic effects and $\sigma_{E}^{2}$ the environmental variance.

Linear regressions between visual and digital measurements are presented from raw data, with regression equations calculated using Model II Linear Regression (Ludbrook, 1997, 2012).

## Results

### Proof of Concept

**FIGURE 3:**
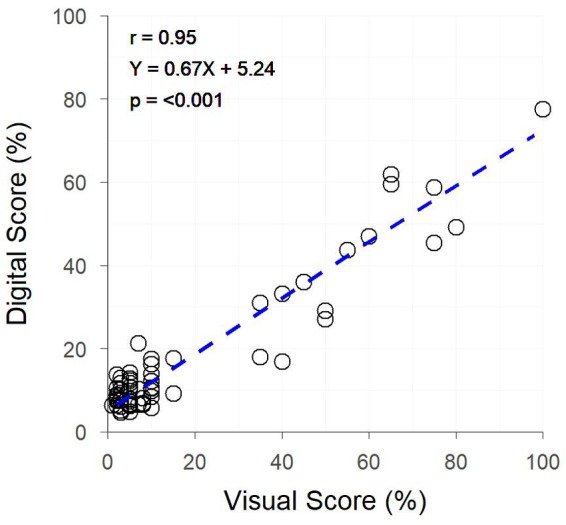
The relationship between Digital Yellow Leaf Area and Visual Physiological Yellowing (PY) scores, for individually potted plants within a Greenhouse. Dashed line represents the linear regression between measurements.

The image analysis methods proposed in Supplementary Data 1 were able to efficiently and consistently segment both plant material from the background image, and yellow plant material from total plant material (Figure 1 top). Digital scores correlated strongly (*r* = 0.95) with visual scores assessed from individual plants (Figure 3), with genetic and residual correlations being similarly, strong (Table 2). Heritability for both measurements showed similarly, high values, with visual scores being slightly higher (Table 2).

**Table 2:** The correlation coefficients (r) for raw data, genetic and residual correlations between visual and digital scores, and the heritability (H^2^) of individual data sets collected for the traits physiological yellowing (PY), senescence, Septoria tritici blotch (STB) and canopy cover.

Trait	Trial	Raw Correlation	Genetic Correlation	Residual Correlation	H^2^ – Visual	H^2^ – Digital
Physiological Yellows	*Greenhouse*	0.95	0.98	0.92	0.85	0.76
	*A*	0.61	0.86	0.32	0.46	0.74
	*B*	0.78	0.86	0.66	0.46	0.73
Senescence	*C*	0.74	0.83	0.43	0.76	0.81
	*D*	0.75	0.92	0.44	0.67	0.74
Septoria tritici blotch	*E*	0.76	0.86	0.47	0.73	0.59
Canopy Cover	*F – Time 1 (Z25)*	0.88	0.82	0.82	0.11	0.08
	*F – Time 2 (Z69)*	0.86	0.92	0.75	0.59	0.72

### RGB Imaging in Field Conditions

Following the success of applying the proposed image analysis methods to individual plants in a greenhouse environment, hand-held images of field plots were collected to further test the application of the methods and investigate their robustness under field conditions. As with greenhouse images, the image analysis methods proposed in the current study were capable of segmenting plant and background pixels, in this case from soil rather than a plain background, as well as separating yellow plant material from total plant material.

**FIGURE 4:**
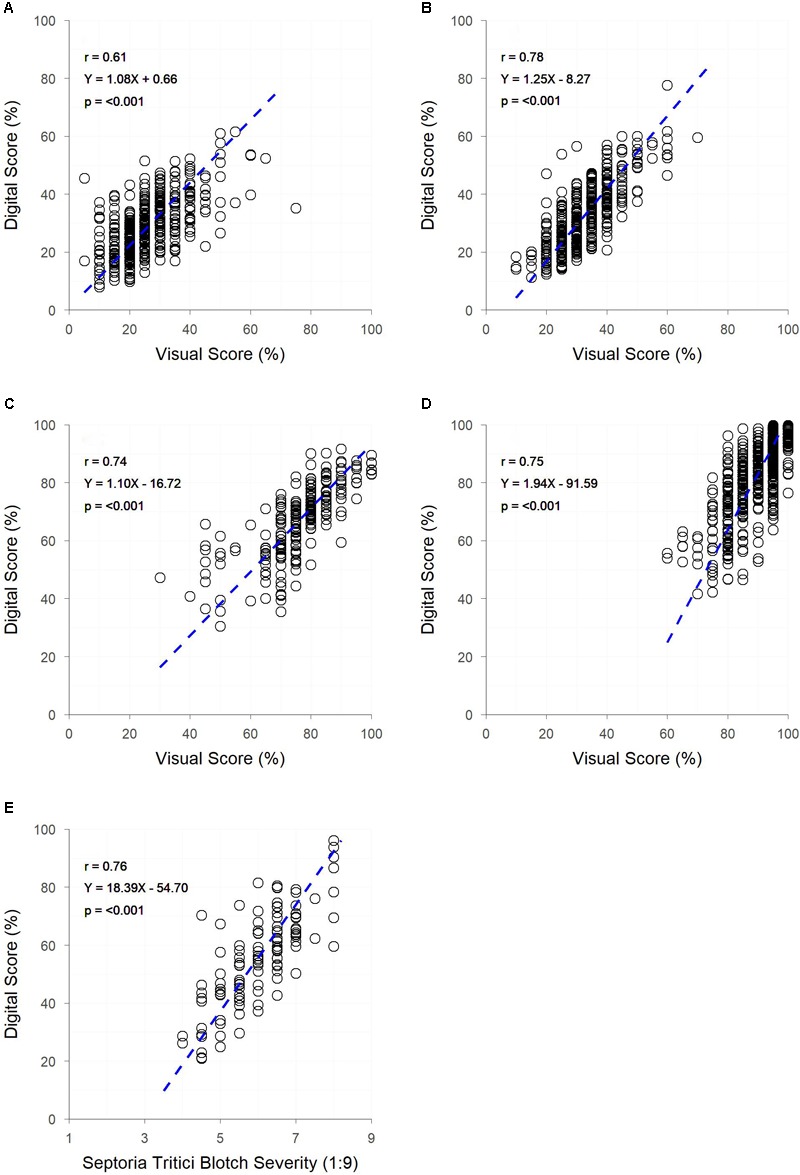
The relationships between visual and digital scores for digital yellow leaf area and visual PY score in field trials **(A,B)** digital yellow leaf area and visual senescence score for field trials **(C,D)** and digital yellow leaf area and visual Septoria tritici blotch (STB) severity score for field trial **(E)**. Dashed lines represent the linear regression between measurements.

Significant correlations (*p* < 0.001) were observed between digital and visual scores across all field trials (Figure 4). A slightly weaker correlation between visual and digital scores was observed in trial A (*r* = 0.61), with trials B, C, D, and E having slightly stronger correlations (*r* = 0.74 – 0.78). For each trial, genetic correlations were stronger than raw correlations between visual and digital measurements, with residual correlations being smaller than raw correlations. For all but trial E, the heritability of the digital score was higher than that of the visual score (Table 2). This was particularly the case for trials A and B where the digital scores had heritability 0.28 and 0.27 units higher than the respective visual scores.

Images were collected by hand at a rate of approximately one image every four seconds across all field trials, or approximately 900 plots per hour. Image analysis took approximately 10 min per trial, with the bulk of this time spent finessing threshold values. Computer processing time was approximately 0.02 sec per image (0.12 sec per image when including the process of importing images to FiJI). Visual scores (Trials A–E) took over double that time, with one score recorded approximately every nine seconds or 400 plots per hour.

### Deploying Digital Phenotyping Methods on a High-Throughput Infrastructure

The final step in the current study was to deploy the digital phenotyping methods (Supplementary Data 1) on high-throughput infrastructure designed to work effectively within a field-crop breeding programme. Advanced yield plots were imaged using the HIB to assess canopy cover.

**FIGURE 5:**
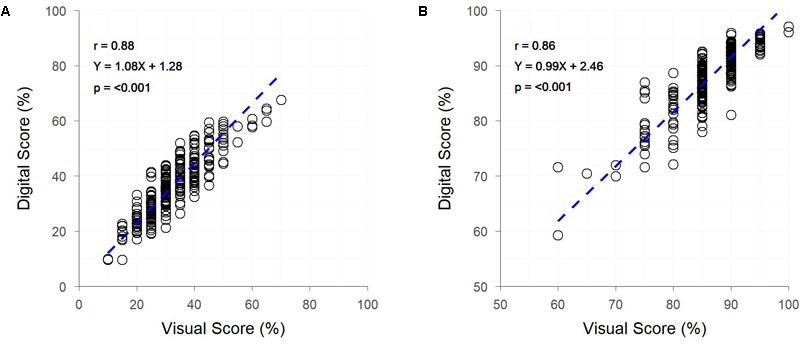
The relationships between visual and digital scores for percent image area green and visual assessment of percentage canopy cover, for field trial F at two time-points – Zadoks Growth Scale 25 **(A)** and 69 **(B)**. Dashed lines represent the linear regression between measurements.

Both early and late assessments of canopy cover (approximately Z25 and Z69, respectively) showed strong correlations between digital and visual scores (*r* = 0.88 and 0.86, respectively) (Figure 5). Early assessment of canopy cover produced genetic and residual correlations of equal strength, both of which were slightly weaker than the raw correlation, though for assessment at Z69 genetic and residual correlations were, respectively, stronger and weaker than the raw correlation (Figure 5). Heritabilities were low for digital and visual scores at Z25, though slightly higher for visual scores, but greatly increased at Z69, with the digital score having a greater heritability than the visual score (Table 2).

The HIB achieved a throughput of approximately 7,400 plots per hour, with the 9,600 plot trial site containing trial F being imaged in 80 min; equating to approximately two unique images per second. Analysis of plot images took approximately 10 min. Accurate *in situ* visual assessment of canopy cover is challenging due to the oblique perspective of the scorer, however, a throughput of approximately 400 plots per hour would be expected, based on scoring rate of other traits in the current study.

## Discussion

Image analysis as a phenotyping tool is a common practise within greenhouse and controlled environment experiments, and a number of commercial platforms and facilities offer streamlined approaches for data collection and analysis (for example the LemnaTec Scanalyzer^1^, and the Australian Plant Phenomics Facility^2^). These systems allow the collection of high temporal resolution data with ease, and are commonly used for the assessment of green leaf area, and subsequently for the assessment other traits such as of the rate of senescence (Atieno et al., 2017). However, these systems are expensive to establish and are limited to assessment of plants grown in controlled environments within pots.

The image analysis methods proposed in the current study offer a low-budget, open-source alternative to the controlled environment systems described above, and are suitable for the collection of digital scores comparable to visual scores of colour-based traits. The example presented in the current study shows the application of these methods to PY. However, as shown by the results of Objectives 2 and 3, these methods are robust across other colour-based traits. The strong correlation between digital and visual assessments of PY in the greenhouse experiment is unsurprising, as the imaging of individual plants in front of a uniform white background provides ideal conditions to implement this type of image analysis. There is little occlusion present, and plant material pixels can be easily segmented within the images due to the vastly different hue values of plant and background material pixels. Despite these ideal conditions, there are limitations to the use of the proposed methods for assessing colour-based traits which do not express uniformly across all plant organs, as the proposed methods are basic and not capable of isolating individual plant organs for analysis. In the case of the current study, stems and ears of plants often remained green while leaves expressed PY, resulting in images still containing a many green pixels. This ultimately reduced the percentage of the plant classed as yellow, leading to a slope <1 for the linear regression between visual and digital scores (Figure 3). Regardless of this limitation, the high heritability of PY for both digital and visual scores in the greenhouse experiment demonstrated the accuracy that is achievable under ideal conditions.

Despite the high-quality data obtainable under controlled conditions, field phenotyping is favoured within plant breeding programmes, to gain an understanding of genotype performance when subject to realistic and relevant environmental conditions and to examine genotype-by-environment interactions (Araus and Cairns, 2014). In contrast to controlled environment imaging, field imaging occurs under conditions that are far from ideal. The main contributing factors to this being the large amount of occlusion which occurs within the crop canopy, preventing plant material in the lower canopy from being fully visible (Casadesús et al., 2007), and the potential for plant pixels and soil pixels to have similar hue values, resulting in a more difficult segmentation process. Despite these limitations, there are still strong similarities between image analysis of greenhouse and field images, as can be seen in the results of Objectives 1 and 2 in the current study. In the case of PY, where images were obtained from both greenhouse and field trials (A and B), direct comparisons can be made around the quality of data collected. While the strongest correlation between digital and visual data was observed in the glasshouse experiment, the heritability of digital and visual scores was similar. Weaker correlations were observed between digital and visual scores within field trials, though the heritability of digital scores was generally greater than for visual scores, indicating that digital scores provide a more accurate assessment of the trait.

The ability to apply the image analysis methods of the current study to a range of traits across multiple field trials demonstrates the robustness of these simple methods. For each field trial (A–F) positive relationships were observed between digital and visual scores, irrespective of the trait, with digital scores generally resulting in a similar or improved heritability compared to visual scores (Table 2). The high heritability of all traits assessed digitally (excluding canopy cover at Z25) indicates the potential to achieve genetic gain through selection for or against the trait. Though heritability of canopy cover was low at Z25, this does not necessarily mean that genetic gain cannot be made for early canopy cover. The low heritability observed in the current study can likely be explained by the variable germination and establishment of plots within the trial, a result of variable soil and poor environmental conditions. These conditions resulted in canopy cover scores being driven by equal levels of genetic and residual variation, leading to a low heritability (Table 2). The potential to achieve genetic gain through selection in these traits is further supported by the relationship observed between raw, genetic and residual correlations, where the raw correlation is not driven purely by the residual, for any of the traits observed. In each instance (excluding canopy cover at Z25) the genetic correlation is greater than the raw and residual correlation, with the residual correlation being weaker than the raw. In trials where residual correlations were high, residuals could be fitted as co-variates within breeding analyses to better model non-genetic effects within the trial. Whether investigating the genetics, or accounting for residual effects in trait performance, the results of the current study show that digital methods can be exchanged with visual methods, while producing greater or maintaining similar heritability. The lower heritability of digital scores, compared to visual scores, observed in trial E is likely a result of (i) the amount of STB occluded from the camera sensor – as the pathogen is spread from the bottom of the canopy up, through rain-splash (Steinberg, 2015), and (ii) patches of senesced grass weeds within the plots. The presence of weeds has likely contributed to the lower heritability in the digital scores of trial F at Z25, with small broadleaf weeds being present in images and contributing to the amount of green pixels present. In both trials E and F visual scores can easily account for occluded leaves or the presence of weeds, which will result in a higher estimate of heritability.

Few studies have compared digital image analysis scores with visual scores of the same trait, opting instead for comparisons to sensor produced visual indices or alternative traits (Adamsen et al., 1999; Lukina et al., 1999; Casadesús et al., 2007; Li et al., 2010; Liu and Pattey, 2010; Mullan and Reynolds, 2010; Kipp et al., 2014). However, direct comparisons between digital and visual scores have been made by Hafsi et al. (2000) and Stewart and McDonald (2014), where individual leaves were isolated on a plain background to obtain images and visual scores. In each of these studies digital scores were found to be effective at estimating the trait of interest (senescence and STB, respectively), corresponding to the results of the current study.

It should be noted that the studies mentioned above used a variety of image analysis methods, some similar to the current study, using thresholds and/or segmentation (Lukina et al., 1999; Li et al., 2010; Liu and Pattey, 2010; Mullan and Reynolds, 2010; Kipp et al., 2014; Stewart and McDonald, 2014). Others have used numerical approaches across the whole image, looking at pixel colour values and ratios (Adamsen et al., 1999; Hafsi et al., 2000; Casadesús et al., 2007).

Despite the variety in previously described methods, image analysis within field experiments is currently far from common practise, with relatively few examples within the literature. Perhaps the most extensive example of using image analysis within large field trials, as well as in the context of plant breeding, is presented by Mullan and Reynolds (2010) where four bread wheat populations were repeatedly imaged and analysed to provide canopy cover values over time. A further example presented by Kipp et al. (2014) showed image analysis to be a superior method of early vigour assessment, compared to spectral sensing. The subject of image collection and processing time was raised in each of these studies, with Mullan and Reynolds (2010) stating an imaging rate of approximately one image every five seconds and an image processing rate of approximately three images per second. Kipp et al. (2014), on the other hand, merely state that their image collection and processing methods are too time consuming for application to large-scale field trials. The processes of image collection and analysis in the current study were conducted in similar times to those reported by Mullan and Reynolds (2010). The combination of image capture and analysis showed a time advantage over visual scores from field trials A–E in the current study, in which images were collected using a hand-held camera, with image collection taking approximately half the time of visual scoring and image processing taking approximately 10 min per trial. This shows that even in the absence of high-throughput methods, digital imaging can save time when scoring breeding or research experiments.

To be adopted by plant breeding programmes, or by large-scale research in general, image analysis methods should be highly automated. This has previously been acknowledged by Casadesús et al. (2007) when investigating digital image analysis for the derivation of visual indices, and is often satisfied through batch processing of images. This was the approach taken in the methods of the current study, greatly reducing the user input required. Further reducing user input could be achieved through the scripting of certain processing steps, however, manual input is still required to correctly apply thresholds to a new set of images. To apply true automation to this process avenues of computer vision and machine learning would need to be explored, such as in the work of Guo et al. (2017), however, such work requires a highly specialised skill set to undertake.

Further to the requirements of automated data processing, high-throughput data collection methods are essential. Platforms for the high-throughput collection of field phenotypic data have been proposed in the literature (Busemeyer et al., 2013; White and Conley, 2013; Andrade-Sanchez et al., 2014; Deery et al., 2014; Bai et al., 2016; Underwood et al., 2017; Jimenez-Berni et al., 2018), though there are currently limited commercial options available. As it stands, a platform that is affordable, truly high-throughput, and easily integrated into large scale breeding and research operations, has yet to be produced.

**FIGURE 6:**
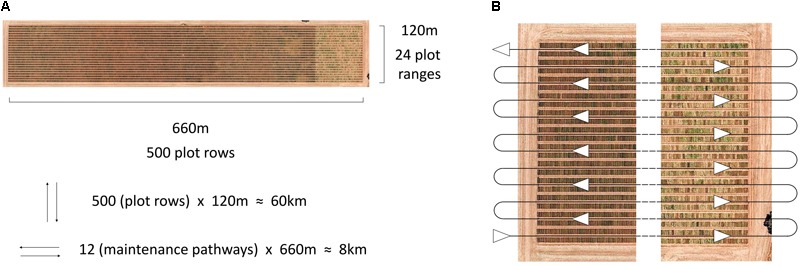
Aerial image of an Australian large-scale wheat breeding site, annotated with dimensions of the site and the number of plot rows, plot ranges and travel distances based on direction **(A)**, and the serpentine path along site maintenance pathways, travelled by the HIB in the current study, allowing for two plots to be imaged simultaneously **(B)**. Image: Google, 2017, Digital Globe.

The HIB used in the current study shows great potential for future deployment within large scale research and plant breeding programmes, meeting the requirements of affordability, high throughput and ease of integration into current trial operations. Traditionally high-throughput phenotyping platforms described in the literature have travelled directly over plots (Busemeyer et al., 2013; White and Conley, 2013; Andrade-Sanchez et al., 2014; Deery et al., 2014; Bai et al., 2016; Underwood et al., 2017; Jimenez-Berni et al., 2018), following the direction of seeding. This allows for thorough data collection over the entire plot, whether multiple images of the canopy or other sensor data, though it greatly increases driving distance and is difficult to implement within standard breeding trials. This is illustrated in Figure 6, where travel along individual plot rows is nearly eight times the distance of travelling along field maintenance tracks, where two plots are imaged simultaneously (one either side of the pathway), when traversing a large-scale field trial in a wheat breeding programme.

Travelling along maintenance pathways within the field trial also offers the benefit of integrating with current field maintenance practises and can take advantage of tractor RTK GPS autosteer profiles that have previously been generated for the maintenance of trial sites. In the current study, GPS coordinates and output from the tractor’s RTK GPS autosteer system were used to automatically trigger image capture. This allowed a “hands-off” data collection approach, as well as ensuring that repeated imaging occurred in the same position for each plot, with a 2 cm tolerance for error. Further to this, the use of autosteer reduces the chance of operator error, assisting in the prevention of accidental damage to field trials.

The small tolerance for error within the image capture system will allow for the extension of this system to earlier stages of the breeding programme, which is often grown in small plots or individual plant rows (Halloran et al., 1979). As demonstrated by the STB images in the current study, the image analysis methods proposed are suitable for application to small plots and are likely transferrable to single rows and potentially single plants. This will be of great interest to plant breeders who wish to conduct phenotypic selection within the early generations of their breeding programme.

At the speed of 5 km/h driven in the current study it was possible to image approximately 7,400 plots per hour. While this is already exceptionally high throughput, the system is capable of operating at higher speeds (with 10 km/h successfully tested). At higher speeds movement is introduced into the boom arms when travelling on uneven ground, and can result in plot images being off-centre. However, these issues could be easily addressed through modification to the boom or tractor, for example, auto-levelling boom arms or lower tractor tyre pressure to reduce boom arm and camera movement. The throughput of imaging observed in the current study becomes even more impressive when compared against the throughput of other systems. Recent work by Khan et al. (2018) compared the throughput of plot level RGB imaging from two systems; a low-cost Mobile Ground Platform (MGP) and an Unmanned Aerial Vehicle (UAV). In their study, throughputs of 120 plots per hour and 1200 plots per hour were achieved for the MGP and UAV, respectively. While these results show a clear advantage in the throughput of UAVs compared to ground platforms, the throughput achieved by the HIB in the current study is over six times greater than that achieved by Khan et al. (2018) with a UAV. This demonstrates that truly high-throughput ground based, plot level, imaging is achievable and, as described by Khan et al. (2018), can deliver high-fidelity images of crop canopies not currently achievable with UAVs.

Deployment of the HIB within a large-scale wheat breeding programme during the 2017 growing season allowed for images of individual plots to be captured with extremely high throughput. While data from a single site is presented for the assessment of canopy cover in the current study, the system was deployed at eight trial sites across southern Australia and used to collect 288,680 images from 74,880 unique field plots. Images acquired with the HIB are suitable for the application of the image analysis methods proposed in the current study, enabling wheat breeders to efficiently and objectively assess colour-based traits.

Though the current study has focused on images collected from RGB cameras mounted on the platform, it is possible to expand the system for the collection of a greater variety of data. Numerous sensors have been proposed as high-throughput field phenotyping tools, such as LiDAR (Deery et al., 2014; Bai et al., 2016; Underwood et al., 2017; Jimenez-Berni et al., 2018), multispectral and hyperspectral cameras (Busemeyer et al., 2013; Bai et al., 2016; Underwood et al., 2017), thermal sensors/cameras (Crain et al., 2016; Deery et al., 2016) and NDVI (Bai et al., 2016; Crain et al., 2016; Underwood et al., 2017), all of which could be integrated to the HIB.

## Conclusion

The basic image analysis methods described in the current study are effectively able to produce digital scores that correlate well to visual scores for colour-based traits, with examples being presented for PY, senescence, STB and canopy cover. The methods described in the current study have a low barrier to entry and utilise commercially available digital cameras and open-source computer software. This, combined with the strong correlations observed between digital and visual data, the high heritability of assessments, and the associated time savings, make for an attractive set of methods for the assessment of colour-based traits within a wheat breeding programme. Furthermore, they show potential for application within other breeding programmes, particularly other cereals and field crops.

To further encourage the adoption of image analysis within plant breeding programmes, an effective system for the high-throughput collection of images has been described, including a clear pathway for integration into current field maintenance practises. This system was deployed within a wheat breeding programme and is capable of high-throughput large-scale image collection, providing images suitable for the analysis methods described in the current study. This ultimately provides a rapid and objective data collection methodology, enabling unprecedented levels of data collection from large-scale plant breeding field trials.

There is further potential to increase the value of collected images to breeding programmes, through the implementation of more complex image analysis methods – focusing on other applications such as seedling counting (Liu et al., 2016), ear counting and flowering detection (Sadeghi-Tehran et al., 2017; Virlet et al., 2017). High-throughput collection and processing of such data, from large-scale field trials, will only further strengthen the role of image analysis within plant breeding programmes.

## Author Contributions

JW, JE, JC, HK, and SM designed the High-Throughput Image Boom (HIB). JC wrote the image acquisition software with input from JW, JE, SM, and HK. JE and HK designed the experiments and managed the wheat breeding programme. JW established the data processing methods, and collected and analysed all the data. JW, JE, GM, and HK interpreted the analysed data. JW wrote the manuscript with contributions from JE, JC, GM, SM, and HK.

## Conflict of Interest Statement

JW, JE, and HK are affiliated with Australian Grain Technologies Pty Ltd, a commercial plant breeding company. The remaining authors declare that the research was conducted in the absence of any commercial or financial relationships that could be construed as a potential conflict of interest.

## Footnotes

1 https://www.lemnatec.com/products/high-throughput-phenotyping-solutions/greenhouse-scanalyzer/

2 https://www.plantphenomics.org.au/
