# Supplementary material for: High-Throughput Field Imaging and Basic Image Analysis in a Wheat Breeding Programme
Source: Front Plant Sci. 2019 Apr 24;10:449. doi: 10.3389/fpls.2019.00449 (PMC6492763; doi:10.3389/fpls.2019.00449)
Supplement: Supplementary file 1 [file Data_Sheet_1.PDF]

Supplementary Material

Basic Colour Segmentation of Digital Images

Using:

FiJI – https://fiji.sc/

Threshold_Colour Plugin – http://www.mecourse.com/landinig/software/software.html

1. Import images as a stack

- File > Import > Image Sequence…
- Navigate to the folder containing your images and select the first image

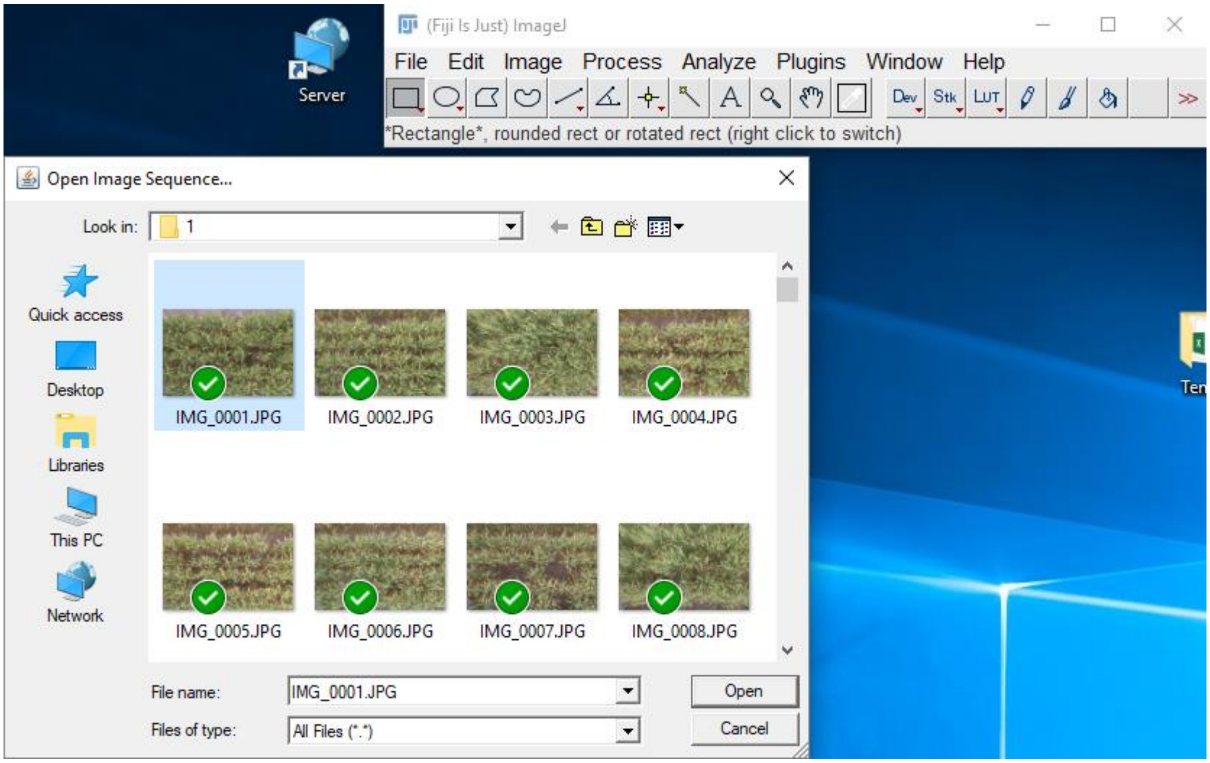

- Specify number of images (if default is incorrect) in the Sequence Options Window*
    - *Depending on RAM limitations of the computer, images may need to be resized before loaded into image-J, especially when dealing with a large number of files.

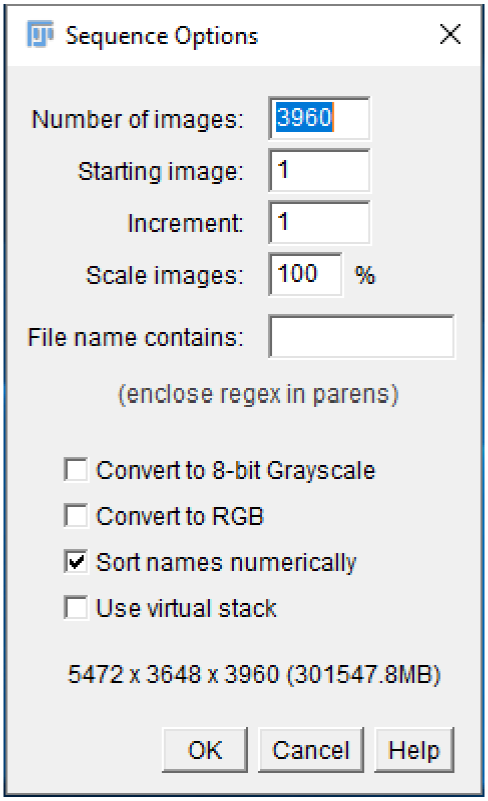

2. Define Regions of Interest (ROI) in images (if required)

- Analyze > Tools > ROI manager
- Using the cursor, select the area of your image you wish to perform the analysis on*

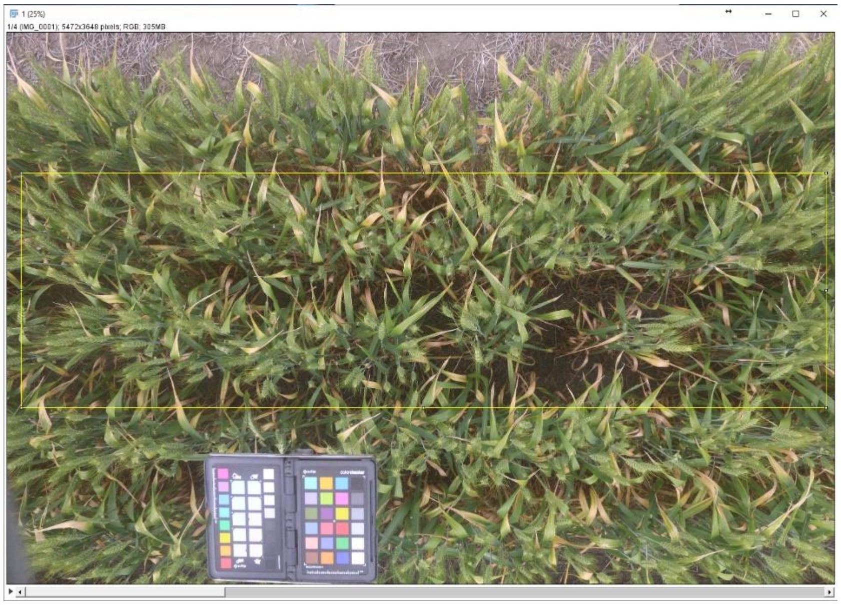

- Once the area is selected, press the 'Add' button in the ROI manager
  - *You can cycle through images using the scroll bar at the bottom, to ensure the ROI is suitable across your image set

3. Convert images to ROI stack

- Ensure that the ROI you have created is selected (highlighted) in the ROI manager

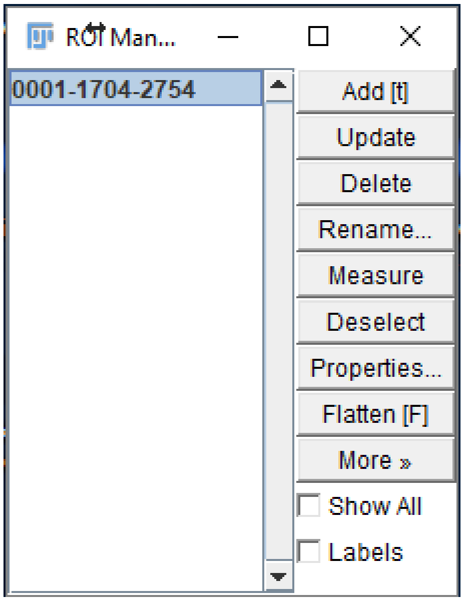

- Image > Duplicate (make sure the 'Duplicate stack' box is checked)

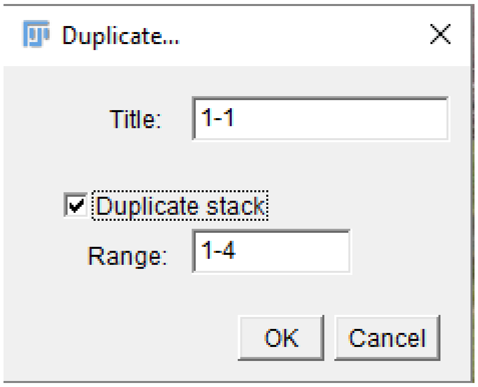

- A new window will appear with all the ROI from your image stack. This may take a moment to process, especially for large image sets
- At this point, the original image stack and the ROI manager window can be closed (if desired)
- The ROI image stack can also be saved by navigating to File > Save As > Image sequence

4. Duplicate the ROI stack

- Duplicate the ROI image stack, one stack will be used for measuring Total Leaf Area, while the other will measure Leaf Area Yellow
- Image > Duplicate (make sure the 'Duplicate stack' box is checked)
- A second window containing the ROI stack will appear

5. Threshold for Total Leaf Area

- Select one of the ROI stacks
- Navigate to Plugins > Threshold Colour > Threshold Colour
- Use the scroll bars in the Threshold Colour window to select the desired threshold
- Hue values should remove reds and blues from the image, keeping yellows and greens. This will likely be in the range of 15 – 100
- Thresholding on Brightness may be useful to remove dark and shadowy patches of soil, but it is important not to remove large areas of dark leaves.
- Saturation values may or may not be useful, depending on the situation.
- This stage needs to be tweaked for each set of images analysed, taking into account soil colour and illumination of the image.

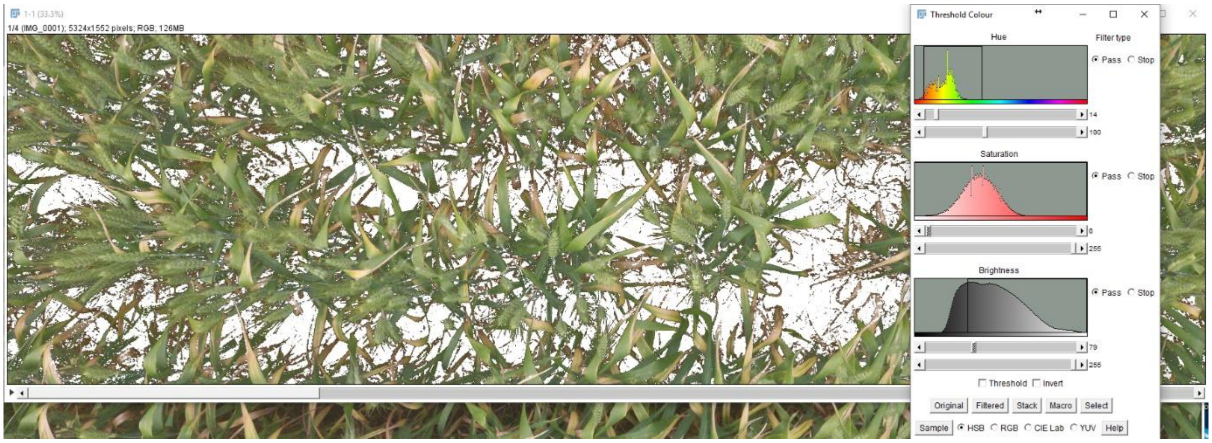

- Press the ‘Stack’ button to apply these selections to all images and check they are suitable
- Once a suitable threshold has been created, check the ‘Threshold’ box and then press the ‘Stack button’ to apply a black and white threshold across all images.

6. Convert to binary image

- Process > Binary > Make Binary
- In the Convert Stack to Binary window, use the settings from the below figure

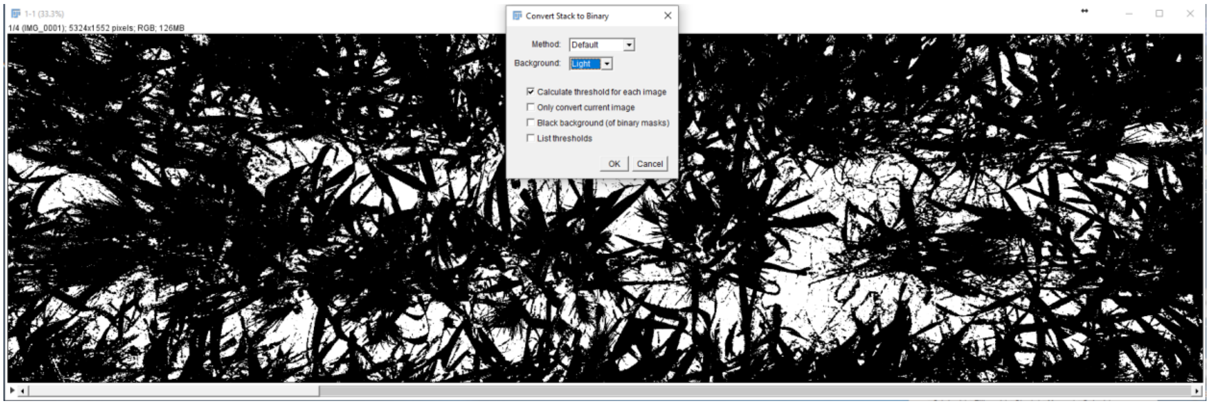

7. Count Total Plant Area Pixels

- Plugins > Voxel Counter
- This will give the number of black pixels per image i.e. the number of plant pixels
- Save data from the results window, or copy to the desired document.

9. Threshold for Yellow Leaf Area

- Select the unprocessed image stack, created in step 4
- Repeat steps 5, 6 and 7. In step 5 instead of thresholding for greens and yellows, threshold for the yellow colour of interest.
- This will likely be in the Hue region of 15-35

10. Calculate percentage of Total Leaf Area and Leaf Area Yellow

- Use (Yellow Leaf Area Pixels/Total Leaf Area Pixels)*100 to get a percentage score of Yellow Leaf Area
- Percentage canopy cover can also be calculated as (Total Leaf Area Pixels/Total Pixels in ROI)*100
