# Supplementary material for: High-Throughput Field Imaging and Basic Image Analysis in a Wheat Breeding Programme
Source: Front Plant Sci. 2019 Apr 24;10:449. doi: 10.3389/fpls.2019.00449 (PMC6492763; doi:10.3389/fpls.2019.00449)
Supplement: Supplementary file 2 [file Data_Sheet_2.PDF]

Supplementary Material

Image processing examples for Senescence, Septoria Tritici Blotch Severity and Canopy Cover

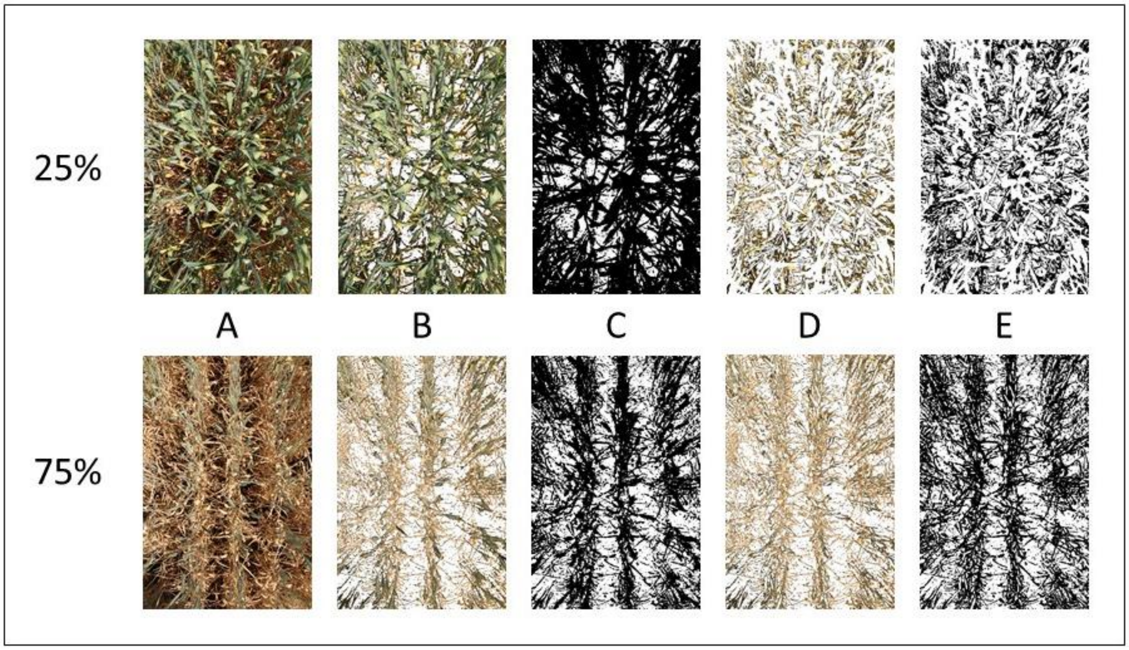

Supplementary Figure 1. Examples of images processed for the assessment of senescence, showing the original image **(A)**, segmented plant material **(B)**, binary plant material threshold **(C)**, segmented senesced material **(D)** and binary senesced material threshold **(E)**, for plots with scored approximately **25%** and **75%** yellow leaf area.

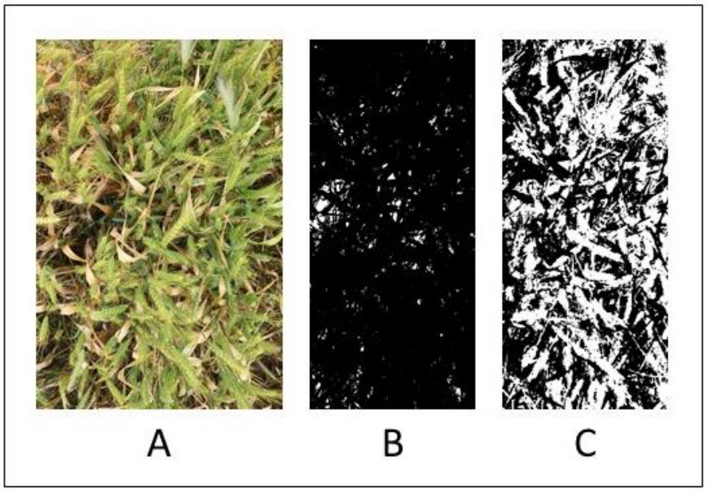

Supplementary Figure 2. Examples of images processed for Septoria tritici blotch assessment, showing **(A)** original image, **(B)** binary plant material threshold and **(C)** binary Septoria tritici blotch material threshold.

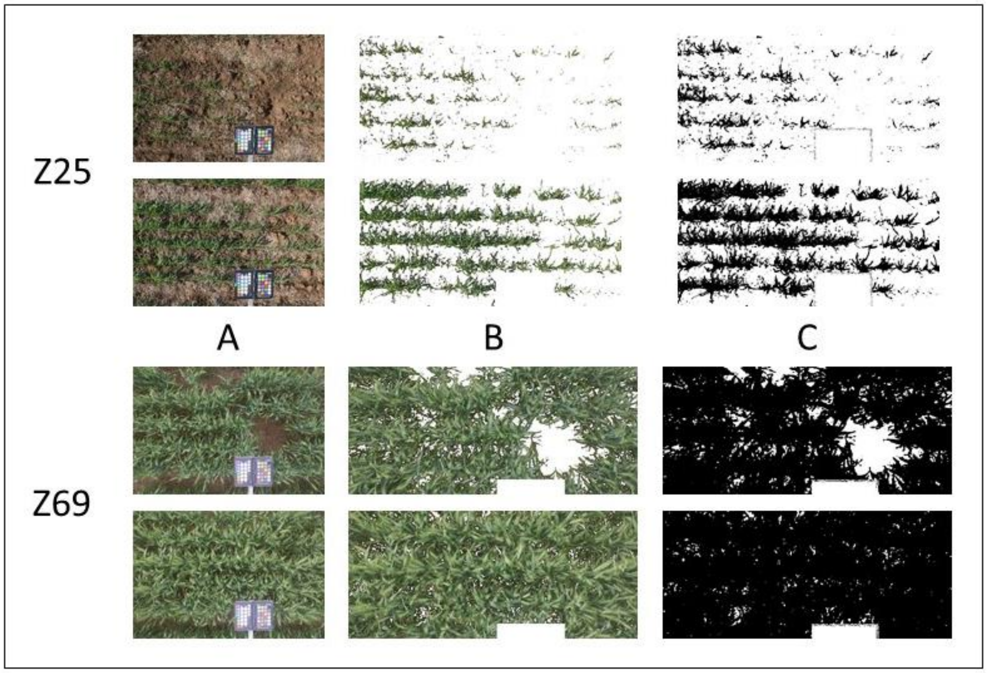

Supplementary Figure 3. Examples of images processed for canopy cover assessment, showing **(A)** original image, **(B)** segmented plant material and **(C)** binary plant material threshold, for plots at **Zadoks growth scale 25 (top)** and **69 (bottom)**.
